# Efficacy of a novel chewable tablet (Credelio Quattro™) containing lotilaner, moxidectin, praziquantel, and pyrantel for the prevention of heartworm disease (*Dirofilaria immitis*) in dogs

**DOI:** 10.1186/s13071-026-07379-0

**Published:** 2026-04-06

**Authors:** Lisa Young, Craig R. Reinemeyer, Mansour Abdelmoneim, Scott Wiseman

**Affiliations:** 1 Elanco Animal Health, 450 Elanco Circle, Indianapolis, IN 46221 USA; 2 East Tennessee Clinical Research, Inc, Rockwood, TN 37854 USA; 3 TRS Labs, Inc., Athens, GA 30607 USA; 4 Elanco Animal Health, Form 2, Bartley Way, Bartley Wood Business Park, Hook, RG27 9XA UK

**Keywords:** Credelio Quattro, *Dirofilaria immitis*, Moxidectin, Heartworm

## Abstract

**Background:**

Canine heartworm (*Dirofilaria immitis*), a filarial parasite endemic in many countries worldwide, can cause life-threatening disease in dogs if left untreated. Two laboratory studies and one field study were conducted to evaluate the efficacy of Credelio Quattro, a novel chewable tablet containing moxidectin in combination with lotilaner, praziquantel and pyrantel, for the prevention of heartworm disease caused by *D. immitis*.

**Methods:**

In the laboratory studies, dogs were inoculated with 50 *D. immitis* L3 larvae on Day – 30. Eight dogs per group were randomized to receive either a placebo or Credelio Quattro at the minimum effective dosage of 0.02 mg/kg moxidectin, 20 mg/kg lotilaner, 5 mg/kg praziquantel, and 5 mg/kg pyrantel on Day 0. One of the studies also included a lotilaner-only treated group to demonstrate non-interference. Efficacy was evaluated ~ 150 days post-inoculation utilizing adult heartworm counts collected at necropsy. The field study enrolled client-owned dogs, ≥ 8 eight weeks of age, weighing at least 1.5 kg, from 13 geographically diverse veterinary clinics throughout the USA. Dogs were randomized in a 1:1 ratio to receive either Credelio Quattro or a positive control product, Simparica Trio^®^ (moxidectin/sarolaner/pyrantel), at the recommended label dosages for 11 consecutive months. Efficacy was evaluated on Day 330 by assessing the detection of adult heartworm infections using antigen and microfilarial tests.

**Results:**

In both laboratory studies, no adult heartworms were collected from any of the dogs treated with a single dose of Credelio Quattro. In contrast, all dogs treated with placebo (range 13–40) or lotilaner-only (range 9–42) had adults present. A total of 372 dogs were enrolled in the field study (191 Credelio Quattro, 181 Simparica Trio). At the end of the study, no dogs from either treatment group tested positive for adult heartworms. Credelio Quattro was well tolerated in all studies.

**Conclusions:**

The laboratory studies demonstrated Credelio Quattro as 100% effective for the prevention of heartworm disease caused by *D. immitis* in dogs. Additionally, no dogs in the field study tested positive for adult heartworm infection after 11 consecutive monthly doses of Credelio Quattro.

**Graphical abstract:**

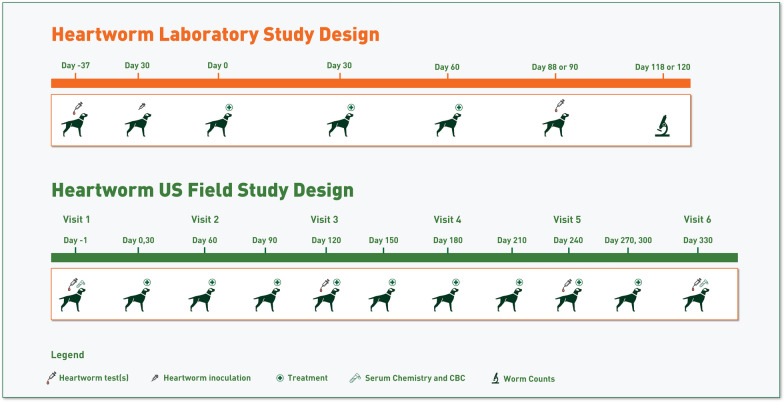

## Background

Canine heartworm (*Dirofilaria immitis*) is a filarial parasite found worldwide and is prevalent in many countries such as Australia, North America, Brazil, and the Mediterranean [1]. Discoveries of canine heartworm were made as early as 1626 in Italy [2], in 1847 in the USA, and in 1875 in South America [3]. Heartworm disease can be prevented by the administration of macrocyclic lactone (ML) heartworm preventive products. Despite recommendations for year-round preventive administration by the American Heartworm Society (AHS) and the Companion Animal Parasite Council (CAPC), the incidence of heartworm disease continues to increase [4]. Over the last several years, lack-of-effectiveness (LOE) claims against approved ML-containing drug products available as heartworm (HW) preventives [5–7] have been reported in the USA. Multiple factors may contribute to LOE, such as poor owner compliance, incorrect dose rate based on body weight of the dog, missed doses, and the development of HW resistance [8].

MLs are the active component of all current heartworm preventive medications and are highly effective against susceptible isolates of *D. immitis* [5]. This class of compounds is derived from soil-dwelling fungi *Streptomyces* and are the only Food and Drug Administration (FDA)-approved heartworm preventive medications currently marketed (ivermectin, milbemycin oxime, moxidectin, and selamectin) [9]. MLs target the third and fourth larval stages (L3, L4) of the heartworm’s life cycle, killing the larvae before development to the adult stages in the pulmonary vasculature. The first MLs approved by the FDA for the prevention of heartworm disease were ivermectin (Heartgard-30^®^, Boehringer Ingelheim, Duluth, GA, USA) in 1987 and milbemycin oxime (Interceptor^®^, Elanco Animal Health, Indianapolis, IN, USA) in 1990 [10]. Against known ML-resistant heartworm isolates, moxidectin has demonstrated superior efficacy to ivermectin and milbemycin oxime when administered at higher doses [11]. Moxidectin is known to be highly lipophilic [12], has a higher tissue distribution than other MLs, and has a longer elimination half-life, which may contribute to moxidectin’s increased potency compared with other MLs [13]. All MLs target the glutamate-gated chloride ion channels (GluCls) in invertebrates and filarial nematodes; however, moxidectin has subtle structural differences, making the binding of moxidectin to nematode GluCls different from that of the other MLs and hence potentially slowing the development of drug resistance to moxidectin [13]. Additionally, moxidectin shows a lower affinity for GABA_A_ channels, demonstrating a wider margin of safety compared with ivermectin [14].

Moxidectin, used as a HW preventive, is currently available in various formulations and is successful against susceptible HW and highly effective against some ML-resistant HW isolates. The efficacy of various moxidectin formulations against susceptible and resistant heartworm has been reviewed previously [13]. Briefly, a topical application of moxidectin 2.5 mg/kg (in combination with 10 mg/kg imidacloprid, Advantage Multi^TM^, Elanco Animal Health) has demonstrated efficacy against infection with ML-resistant JYD-34 heartworm 30 days after a single treatment [6]. An extended-release, injectable moxidectin formulation (ProHeart^®^ 6, Zoetis, Kalamazoo, MI, USA) demonstrated 99.5% efficacy against the ML-resistant JYD-34 isolate when L3 were inoculated 2 days after treatment [15]. Moxidectin, administered orally at 0.024 mg/kg for 3 consecutive months, was 98.8% effective against JYD-34 [13]. The proven efficacy of moxidectin against various HW isolates, both susceptible and ML-resistant, led to the development of an oral tablet formulation that included moxidectin at a minimum dosage of 0.02 mg/kg (range 0.02–0.04 mg/kg) in combination with lotilaner (20–40 mg/kg), pyrantel (5–10 mg/kg), and praziquantel (5–10 mg/kg) for broad-spectrum parasite coverage.

CAPC recommends broad-spectrum parasite control with efficacy against heartworm, intestinal parasites, fleas, and ticks be administered year-round [16]. Ectoparasites such as fleas and ticks not only induce allergic reactions in dogs, but they also act as vectors for other infectious diseases such as ehrlichiosis, anaplasmosis, and babesiosis [17]. Lotilaner is an ectoparasiticide belonging to the isoxazoline drug class [7, 18] and was formulated as a mono-use drug product (Credelio™, Elanco Animal Health) that provides rapid, consistent efficacy against fleas and ticks in both dogs and cats [19–21]. Pyrantel is a member of the tetrahydropyrimidine family, with anthelmintic activity against both immature and adult roundworms and adult hookworms [22–24]. Praziquantel is a well-known anthelmintic that targets cestodes [25–27], providing 100% efficacy against *Dipylidium caninum*, *Taenia pisiformis*, and the zoonotic cestodes, *Echinococcus granulosus*, and *E. multilocularis* [26].

Credelio Quattro, containing a combination of lotilaner, moxidectin, praziquantel, and pyrantel pamoate, was evaluated for its efficacy against a wide range of parasites–heartworm, lungworm, intestinal nematodes [28–30], and cestodes [31], as well as fleas and ticks. Credelio Quattro's convenient monthly chewable tablet provides broad-spectrum efficacy, promoting year-round parasite protection.

Three studies, two laboratory studies and a field study, were conducted to assess the efficacy of Credelio Quattro in the prevention of heartworm disease in dogs. Studies 1 and 2 were laboratory-based clinical studies in which the disease was artificially induced by experimental inoculation. Studies 1 and 2 evaluated the safety and efficacy of the combination tablet, with Study 2 also assessing the non-interference of lotilaner. Study 3 was a field study designed to evaluate the safety and efficacy of the combination tablet, administered orally, once monthly for 11 months in client-owned dogs in geographically diverse heartworm-endemic areas within the USA.

## Methods

### Animal ethics and welfare

The use of animals and all animal procedures were approved by the Elanco Animal Care and Use Committee and the Animal Care and Use Committee of the institution performing the study prior to study initiation. Studies were conducted in accordance with VICH GL9 Good Clinical Practice, VICH GL 19 Effectiveness of Anthelmintics: Specific Recommendations for Canine [32] and VICH GL7 Effectiveness of Anthelmintics: General Recommendations. Informed owner consent was obtained prior to enrollment in the field study.

### Laboratory studies

#### Animals

Study 1 was conducted using 16 male and 16 female purpose-bred Beagle dogs in good health, approximately 7–10 months of age at the time of inoculation, and weighing 6.8–14.8 kg on Day – 1. Study 2 was conducted using 20 male and 20 female Beagle dogs in good health, aged approximately 6–8 months at the time of inoculation, and weighing 7.1–12.0 kg. on Day – 1. In both studies, dogs were housed individually in facilities that complied with animal welfare guidelines. Dogs received an age-appropriate commercial dry dog food and were provided water ad libitum. Health observations were conducted at least once daily throughout the studies.

#### Study design and isolate of *D. immitis*

**Fig. 1:**
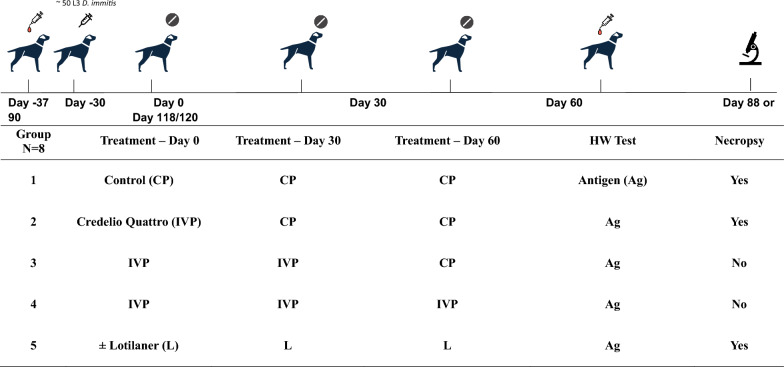
Study design of laboratory Studies 1 and 2, to evaluate the safety and efficacy of Credelio Quattro in preventing heartworm disease

Each study was blinded and randomized, stratified by gender. Dogs were allocated to one of four treatment groups in Study 1 or five treatment groups in Study 2 (Fig. 1), utilizing a consecutive monthly dosing regimen for up to 3 months. Each group included eight dogs, with an equal distribution of males and females. Treatment was administered in a consecutive treatment design such that Group 1 received Control Product (CP) on Days 0, 30, and 60; Group 2 received Credelio Quattro (IVP) on Day 0 and CP on Days 30 and 60; Group 3 received IVP on Days 0 and 30 and CP on Day 60; Group 4 received IVP on Days 0, 30, and 60; Group 5 (Study 2 only) received lotilaner on Days 0, 30, and 60 (refer to Fig. 1). All dogs were inoculated with *D. immitis* on Day – 30, and experimental treatment began on Day 0. Necropsies were conducted on Day 120 (151 days post-infection; Study 1, Groups 1 and 2) and 118 (149 days post-infection; Study 2, Groups 1, 2, and 5).

The *D. immitis* isolates used were obtained from heartworm-endemic areas in the southeastern USA. They were isolated from infected dogs in the field within 5 years prior to study in-life. Study 1 utilized Georgia III (GA-3), originating in Oconee County, Geogia, USA, and Study 2 utilized the SC-20 isolate, originating in Greenwood, South Carolina. At the time of study conduct, neither isolate had been characterized for susceptibility. However, newly published data indicate that SC-20 is a susceptible isolate and GA-3 is considered a putative ML susceptible isolate [33–35]. The GA-3 isolate was demonstrated to contain a genetically mixed population of both susceptible and resistant genotypes. Published data conclude that this isolate may behave as ML susceptible but may be developing resistance [34, 35]. Fifty L3 *D. immitis* were subcutaneously inoculated in the inguinal region on Day – 30.

#### Treatment

On Days 0, 30, and 60, dogs in Study 1 and 2 were administered placebo CP tablets, oral flavored chewable combination tablets (IVP), or lotilaner only (L) (Study 2 only). Dogs were dosed with the appropriate combination of tablets to provide as close as possible to the minimum effective dosage (lotilaner 20 mg/kg, moxidectin 0.02 mg/kg, praziquantel 5.0 mg/kg, pyrantel 5.0 mg/kg), relative to the combination or lotilaner-alone tablet.

#### Heartworm evaluation

In Studies 1 and 2, blood samples from each dog were collected on Days – 35 and – 32, respectively. The blood was evaluated to confirm the absence of adult *D. immitis* antigen (DiroCHEK^®^, Zoetis, Kalamazoo, MI, USA) and to verify the absence of circulating *D. immitis* microfilariae (MF) (modified Knott’s test), conducted as previously described [32, 36] prior to experimental inoculation. On Days 88 (Study 1) and 90 (Study 2), blood samples from each dog were collected and evaluated for the presence of adult *D. immitis* antigen to confirm the dogs did not have an undetected heartworm infection by diagnostic testing prior to inoculation.

Dogs in Study 1 (Groups 1 and 2) and Study 2 (Groups 1, 2 and 5) were humanely euthanized on Days 120 and 118, respectively. After euthanasia, the inoculation injection site (inguinal area) was incised, dissected, and examined for heartworms. The abdominal and thoracic cavities were examined for adult *D. immitis* worms. The anterior and caudal vena cavae were clamped with forceps and the heart and lungs removed as a unit. The venae cava, precava, right atrium, right ventricle, and pulmonary arteries were dissected and examined for heartworms. For each dog, the recovered heartworms were sexed and enumerated.

#### Statistical analysis

All statistical analyses were performed using SAS version 9.4 (SAS Institute, Cary, NC, USA), with the individual dog being the experimental unit. A minimum of five worms was required in each of at least six control dogs (Group 1) to show adequate infection. A 100% prevention rate of adult *D. immitis* infections for each IVP treatment group was required to establish the effectiveness of the treatment.

For Studies 1 and 2, to calculate geometric means (GM), the *D. immitis* counts were transformed using the natural logarithm of count plus one [ln (count + 1)]. The treatment group arithmetic mean (AM) of the transformed counts (denoted by mean*) were then inverse-transformed [exp(mean*) – 1] to obtain the GM.

Percent efficacy (%_Efficacy) was calculated as:

$$\% \_{\text{Efficacy }} = { 1}00 \, \times \, \left( {{\mathrm{GM}}_{{{\mathrm{CTL}}}} {-}{\text{ GM}}_{{{\mathrm{TRT}}}} } \right)/{\mathrm{GM}}_{{{\mathrm{CTL}}}}$$

where GM_CTL_ and GM_TRT_ represent the GM worm counts for the control and treatment groups, respectively.

### Field study

**Fig. 2:**
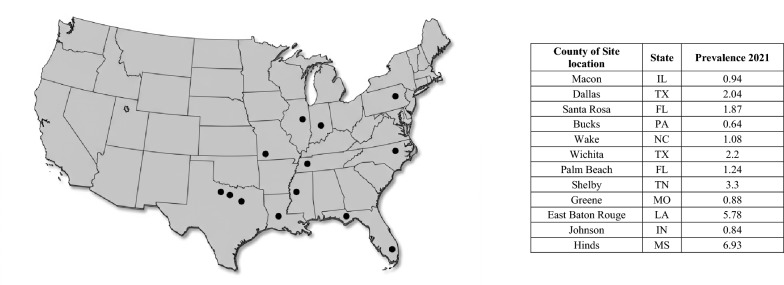
US clinical field study veterinary clinic locations and associated county heartworm prevalence reported by CAPC in 2021

The USA clinical field study was conducted in client-owned dogs in 13 geographically diverse veterinary clinics (Fig. 2) across the eastern and central USA, including sites from the Southeastern USA, the Mississippi Delta region, and the Mississippi River Valley region. Over half of the enrolling clinics were in heartworm-endemic areas, and dog enrollment was initiated during the peak months of HW transmission to provide maximal exposure to mosquitoes that transmit HW. To highlight the 2021 parasite prevalence for each study location, data were extracted from the CAPC prevalence maps. In 2021, the map reported results from > 17 million heartworm tests from three commercial laboratories in the USA. Despite an estimate that < 30% of total activity is represented in the CAPC data reported, the data serve as a statistically significant proxy for confirmation of local parasite pressure [37]. The resulting prevalence values for each county are summarized in Fig. 2 [37]. The study was a randomized, masked, multi-site clinical trial with Simparica Trio (Zoetis, Kalamazoo, MI, USA) as the positive control. The study complied with Good Clinical Practice guidelines.

#### Animals

Dogs of various breeds, ages, and lifestyles were recruited from veterinary clinics in the USA. One dog per household could be enrolled. Dogs had to be at least 8 weeks of age and weigh ≥ 1.5 kg. Dogs of any breed and sex, reproductively neutered or intact (non-pregnant and non-lactating if female), and not intended for breeding during the study were eligible for enrollment. Other eligibility criteria included the following: the dog was of a suitable temperament; the dog had not been treated with Proheart^®^ 6 within 12 months, or Proheart^®^ 12 (Zoetis, Kalamazoo, MI, USA) within 24 months, prior to Visit 1; the dog was generally healthy based on history and physical examination and expected to survive the 11-month study duration; the dog was free of uncontrolled serious disease that could interfere with the objectives of the study or the dog had chronic disease that was clinically stable with appropriate treatment; and the dog had received a monthly HW preventive medication for ≥ 2 consecutive months before Visit 1, with the most recent treatment administered within 21–30 days before Visit 1. Dogs < 6 months of age who had not started on a HW prevention program were eligible for enrollment with a negative HW antigen test. Dogs ≥ 6 months of age were required to have a negative adult HW antigen test and no circulating microfilariae (including *Dirofilaria* sp. and *Acanthocheilonema* sp.).

#### Study design

Dogs were randomly assigned to two groups: Group 1 (IVP) or Group 2 (positive control product), in a 1:1 ratio, with random blocks of two or four dogs, stratified by household type (single or multi-dog). On the initial screening visit (Visit 1, Day – 1), owners signed an informed consent, and dogs were given a physical examination and had blood collected for hematology [complete blood count (CBC)], clinical chemistry (25 parameters), and HW antigen and microfilaria testing. An unmasked treatment administrator at each clinic dispensed the assigned product to the owner for administration to the dog in the home environment. Credelio Quattro was supplied in five strengths to provide the labeled dosage range of 20–40 mg of lotilaner, 0.020–0.040 mg of moxidectin, 5–10 mg praziquantel, and 5–10 mg pyrantel (as a pamoate salt) per kg of body weight (BW) and was administered with food. Simparica Trio was dispensed and administered per label instructions. Along with the treatment administrator at each clinic, owners were aware of treatment group assignment through product packaging, labeling, and instructions. All other clinic personnel conducting clinical observations, measurements, and procedures related to study treatments were masked to treatment group assignments.

**Fig. 3:**
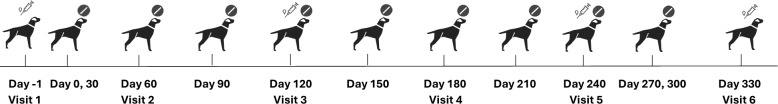
Study design of field study to evaluate the safety and efficacy of Credelio Quattro in preventing heartworm disease

Following Visit 1 (Day – 1), the owner administered the assigned product (IVP or positive control product) for 11 consecutive monthly treatments (targeted treatment days: 0, 30, 60, 90, 120, 150, 180, 210, 240, 270, and 300). Within 3 days of the target treatment date, unmasked clinic personnel confirmed successful tablet administration by pet owners and recorded any observed adverse events. Monthly treatments after the first dose (Day 0) were administered 30 ± 5 days after the previous treatment. Each dog returned to the veterinary clinic and was evaluated on Days 60, 120, 180, 240, and 330. Each visit included a physical examination including BW, heartworm antigen and MF tests (Days 120, 240, and 330), and hematology and serum chemistry (Day 330). Enough assigned product for each dog was dispensed at the respective visit to last until the next scheduled visit (Fig. 3). For all dogs completing the study on Visit 6, MDR-1 genotype testing was conducted. Hematology, clinical chemistries, and HW antigen and modified Knott’s test for MF were conducted at IDEXX Bioanalytics (West Sacramento, CA, USA). Multidrug resistance 1 (MDR1) tests were conducted at Washington State University and reported through IDEXX. Safety of both treatment groups was evaluated through review of abnormal health events, laboratory value changes (hematology, serum chemistry), and changes in BW during the study. Any dog withdrawn from the study prior to completion was included in the safety population but was not evaluated for efficacy.

#### Statistical analysis

All statistical analyses were performed using SAS version 9.4, with the individual dog being the experimental unit. For each dog that completed the study, success or failure of the treatment to prevent heartworm infection was defined by the outcome of the HW antigen and MF tests at the final Day 330 visit. A positive result from either test, confirmed with repeat testing, was considered a treatment failure. For the IVP-treated group, effectiveness was defined as 100% prevention of adult *D. immitis* infections. The HW prevention rate, defined as the percentage of dogs free of adult *D. immitis*, was calculated for each group. Statistical comparison between the IVP-treated and control groups was not performed.

Body weight profiles for the entire duration of the study were investigated using a linear mixed model for repeated measures with treatment, visit, and treatment-by-visit as fixed effects and with site and site-by-treatment as random effects. Baseline body weight was included as a covariate. Treatment groups were compared at each visit, and significant differences were noted at the *p* < 0.05 level. Using the same model, within each treatment group, the change from baseline in body weight at each visit and corresponding 95% confidence interval were also calculated. Baseline body weights were compared in a linear mixed model with treatment as a fixed effect and site and site × treatment as random effects.

## Results

### Laboratory studies

All dogs were dosed, in the fed state, without incident. All dogs tested negative for adult *D. immitis* antigen and MF prior to experimental inoculation and approximately 4 months post-inoculation, confirming that all dogs were negative for *D. immitis* prior to inoculation. On Days 0, 30, and 60, treatment with the combination tablet resulted in dosages in the lower half of the proposed dosing range. Actual dosages ranged from 20.2–26.6 mg/kg for lotilaner, 0.02–0.03 mg/kg for moxidectin, and 5.1–6.8 mg/kg for both pyrantel and praziquantel.

On Day 120 or 118, adult heartworms were recovered from Study 1 and 2, respectively. Adult heartworms were recovered from all eight control dogs in both studies, with worm counts ranging from 13 to 40 and GM of 28.2 and 25.4 in Study 1 and 2, respectively. In Study 2, adult worms were recovered from all eight lotilaner-treated dogs, and worm counts ranged from 9 to 42, with a GM of 29.4. No *D. immitis* worms were recovered from Group 2 (single treatment of IVP) in either Study 1 or Study 2, demonstrating 100% protection from heartworm disease in dogs treated with a single dose of Credelio Quattro (Table 1). As the efficacy of a single dose of the combination tablet was 100%, dogs from Groups 3 and 4 were not euthanized and were returned to the study colony. The GM worm count in the lotilaner-treated group was statistically significantly different (*p* < 0.0001, t_11_ = 21.32) from the zero worms recovered from the IVP-treated group (single treatment).

**Table 1:** Summary statistics of recovered *Dirofilaria immitis* worms

Study	Treatment group (*N* = 8)	GM worm count (range)	Efficacy (%)		Prevention rate (%)	
1 (GA-3)	CP	28.2 (22–36)	n/a		n/a	
	IVP	0.0	100		100	
	Groups 3 and 4	Returned to study colony				
2 (SC-20)	CP	25.4 (13–40)		n/a		n/a
	IVP	0.0		100		100
	Lotilaner	29.4 (9–42)		0		0
	Groups 3 and 4	Returned to study colony				

Treatment with the combination tablet was found to be safe at the dosages administered. No serious adverse events (SAEs) were observed in any group. Non-serious AEs were seen in all treatment groups, including the control. The most common AEs were transient digestive tract disorders (stool abnormalities, diarrhea, regurgitation, and emesis) that resolved without treatment.

### Field study

#### Dog demographics

A total of 396 client-owned dogs were screened across 13 veterinary clinics (Fig. 2) for this field study, and 372 dogs were enrolled between July 19 and August 9, 2021. At enrollment, all dogs were at least 8 weeks of age (range, 2 months–16 years) with the mean age of dogs similar in both groups. Purebred dogs comprised 57.1% and 53.6% of dogs in the Credelio Quattro and positive control groups, respectively. The most enrolled breeds included Labrador Retrievers, Australian Shepherds, German Shepherds, American Pit Bull/Staffordshire Terriers, Golden Retrievers, and Pugs. The demographics of the patients overall were similar across both treatment groups (Table 2).

**Table 2:** Demographics of dogs enrolled in the field study

Demographic		Credelio Quattro (*N* = 191)	Positive control product (*N * = 181)
Gender *n* (%)	Female	90 (47.1)	100 (55.2)
	Female, intact	33 (36.7)	34 (34.0)
	Female, spayed	57 (63.3)	66 (66.0)
	Male	101 (52.9)	81 (44.8)
	Male, intact	37 (36.6)	33 (40.7)
	Male, neutered	64 (63.4)	48 (59.3)
Age (months) at Visit 1	Mean (range)	47.0 (2–194)	43.6 (2–173)
Body weight at Visit 1	Mean (range) kg	20.58 (1.7–86.8)	18.93 (1.5–69.5)
	Mean (range) lbs	45.38 (3.8–191.4)	41.74 (3.4–153.2)
Breed *n* (%)	Mixed breed	81 (42.4)	83 (45.9)
	Purebred	109 (57.1)	97 (53.6)
	Unknown	1 (0.5)	1 (0.6)

#### Evaluable cases

A total of 372 client-owned dogs enrolled in the study received at least one dose of study treatment and underwent safety evaluation. The Credelio Quattro treatment group included 191 dogs, and the positive control product group consisted of 181 dogs. Forty-six dogs (32 in the IVP group and 14 in the positive control product group) withdrew from the study before Day 330. The reasons included owner non-compliance (21 dogs), owner decision (10 dogs), relocation (7 dogs), adverse event unrelated to study treatment (5 dogs), ineligibility (2 dogs), and being difficult to handle (1 dog). Of the 372 dogs in the safety population (SP), 326 dogs completed the study. All dogs that were enrolled, completed the study, and were not observed to have major protocol deviations were evaluated for effectiveness (*n* = 305, Credelio Quattro = 156; positive control group = 149). Twenty-one dogs were excluded from the efficacy evaluation because of various protocol deviations, including study drug overdose (5 dogs) or underdose (4 dogs), missed dose (10 dogs), administration of a prohibited medication (1 dog, Advantage Multi, Elanco Animal Health), and inadvertent unmasking of the Investigator by the owner (1 dog).

#### Heartworm assessment

In the efficacy population, all dogs in both the Credelio Quattro and positive control treatment groups were negative for *D. immitis*, as evidenced by negative results on both HW antigen and MF tests (Table 3) conducted at the final study visit (Day 330). No formal statistical testing regarding heartworm prevention effectiveness was conducted since all dogs in both treatment groups tested negative for HW.

**Table 3:** Summary of heartworm tests in the efficacy population

Treatment group	Study day	Dogs with negative HW tests	% Dogs with negative HW test
Credelio Quattro (*N* = 156)	330	156	100
Simparica Trio (*N* = 149)	330	149	100

#### Safety assessment

The safety population (*n* = 372) included dogs (191 Credelio Quattro, 181 positive control product) receiving at least one dose of treatment. The total number of doses administered in the safety population was 1948 and 1890 in the Credelio Quattro and Simparica Trio groups, respectively. With these doses, five dogs vomited once, within 1 h of dosing. All dogs were redosed successfully, completed the study, and were included in both the safety and efficacy populations.

#### Health observations

Abnormal health events occurred in both treatment groups during the 11-month study period. A total of 51/191 (26.7%) dogs in the IVP-treated group and 30/181 (16.6%) of dogs in the positive control product-treated group experienced abnormal health events. Individual dogs may have experienced more than one clinical sign that was reported as an adverse event. The abnormal clinical signs observed were consistent with those commonly seen in the general dog population, and incidence of AEs occurred at a similar frequency in both treatment groups. Most frequent health events observed in ≥ 2.0% of the dogs in both groups were diarrhea, emesis, lethargy, anorexia, and dermatitis (Table 4).

**Table 4:** Summary of abnormal health events in ≥ 2% of dogs in the study

Clinical sign	Credelio Quattro *N* = 191, *n* (%)	Simparica Trio *N* = 181, *n* (%)
Diarrhea, with or without blood^a^	21 (11.0)	15 (8.3)
Emesis	18 (9.4)	8 (4.4)
Lethargy	12 (6.3)	1 (0.6)
Anorexia	11 (5.8)	5 (2.8)
Dermatitis	10 (5.2)	8 (4.4)
Weight loss	6 (3.1)	3 (1.7)
Alopecia	2 (1.0)	4 (2.2)
Seizure	1 (0.5)	4 (2.2)

^a^Four dogs administered Credelio Quattro and five dogs administered Simparica Trio had bloody diarrhea

Serious abnormal health events occurred in dogs in both treatment groups (10 dogs that received Credelio Quattro; 8 dogs that received Simparica Trio). In the Credelio Quattro group, these included three cases of gastrointestinal foreign body obstruction, one tracheal obstruction, one with bladder stones, one ataxia due to a suspected meningioma diagnosed by a veterinary neurologist, one bilateral pleural effusion, one splenic sarcoma, one tonsillar neoplasia, and one death due to an automobile accident. For the Simparica Trio group, these included one dog with seizures, one with pancreatitis, one with dehydration, one with lethargy, inappetence, and vomiting, one with bladder stones, one with a gastrointestinal foreign body, one with a dog fight injury, and one that died in a car accident. After evaluating clinical examination findings, history, and timing of events, it was concluded that the observed serious adverse events in both treatment groups were unrelated to the study treatment.

The body weight profile in both treatment groups changed at similar rates throughout the study (Table 5). Mean percentage change in BW from Day 0 to Day 330 for the entire SP was 24.5 and 23.4 in the IVP and positive control groups, respectively. Approximately 30% of dogs in each group started the study as juveniles (2–6 months: 46 IVP; 45 positive control; 7–12 months of age: 12 dogs in each group). Both groups had a similar mean BW at Visit 1 (14.2 kg for IVP and 13.6 kg for positive control). Juvenile dogs in the IVP-treated group increased weight by 86.6% by Visit 6, ending the study with a mean BW of 24.5 kg. Juvenile dogs in the positive control product-treated group increased their weight by 78.6%, reaching a mean BW of 20.3 kg by Visit 6. Adult dogs (≥ 12 months of age) started the study at 23.2 and 21.3 kg in the IVP- and positive control product-treated group, respectively. Adult dog BW remained relatively consistent throughout the study. Dogs in the IVP-treated group increased in BW, reaching a mean of 23.9 kg at Visit 6, while dogs in the positive control product-treated group increased, reaching a mean of 21.6 kg at Visit 6. The difference between groups was not statistically significant at either Visit 1 or Visit 6 and was not considered clinically relevant when evaluating the numerous breeds and sizes of dogs in the study.

**Table 5:** Safety population (all dogs): BW, visits 1 and 6

Group	Observed data		Linear mixed model estimates		
	1 (*N* = 191)	2 (*N* = 181)	1 (*N* = 191)	2 (*N* = 181)	*P*-value (test statistic)
Mean BW, Visit 1, kg (CI)	20.6	18.9	20.5 (17.7, 23.3)	18.9 (16.0, 21.7)	0.2399 (*t*_12_ = 1.24)
Mean BW, Visit 6, kg (CI)	23.9	21.6	22.8 (22.0, 23.7)	22.4 (21.6, 23.2)	0.3194 (*t*_1715_ = 1.00)
Mean change, kg (CI)	2.9	2.5	2.9 (2.9, 3.5)	2.6 (2.0, 3.1)	n/a

The hematology results on Day 330 were within the normal reference range and showed no significant patterns or trends related to treatment administration. In general, the serum chemistry findings across both the IVP- and positive control product-treated groups on Day 330 were in line with those of dogs of various ages and breeds receiving standard veterinary care and veterinary medications over an extended duration of study. No consistent patterns of significant changes in serum chemistry were observed throughout the study.

MDR1 testing was performed on dogs completing the study to assess any DNA mutation in the ability to effectively transport the drug, increasing susceptibility to drug toxicosis. Of the 325 tests conducted on the final study visit, 322 dogs had no evidence of a genetic mutation (normal/normal), and three dogs were heterozygous (mutant/normal) for the MDR1 mutation (two in the IVP-treated group and one in the positive control product-treated group). None of the heterozygous dogs experienced adverse events associated with the MDR1 gene mutation.

#### Product acceptability

Credelio Quattro was well accepted by dogs. A total of 561 doses of Credelio Quattro were administered in the safety population. In total, 86.8% of the doses were accepted either by free choice (in food bowl/on floor or by hand) or in food; 58.8% were accepted free choice (the dog had 1 min to consume the tablet from the food bowl or the floor, then another minute to consume the tablet by hand before the tablet was offered in food); and 28.0% were accepted with food.

## Discussion

*Dirofilaria immitis* infection in dogs can be a life-threatening disease with pathological changes occurring secondary to the presence of the adult worms, as well as the result of the impact of thromboemboli caused by dead and dying worms. Clinical signs vary based on the number of worms and chronicity of infection and can range from asymptomatic or mild signs, such as weight loss, exercise intolerance, and lethargy, to more severe clinical signs, progressing to include disseminated intravascular coagulation, pulmonary arterial disease, glomerulonephritis, and potentially caval syndrome [33].

As a preventable parasitic infection with significant morbidity and mortality, prophylaxis is highly preferred to treatment, which can be expensive and is not without risk. Given the severity of heartworm infection, achieving high single-dose efficacy is important for protecting pets, maintaining owner confidence in prevention, and potentially slowing the spread of heartworms, including isolates resistant to MLs [13].

The efficacy of Credelio Quattro in preventing heartworm disease was 100% against experimentally induced infections under laboratory conditions. In the field study, none of the 156 client-owned dogs that were administered monthly doses of Credelio Quattro tested positive for adult *D. immitis* at the conclusion of the study. Laboratory studies demonstrated efficacy with a single dose, and the field study confirmed efficacy in the hands of pet owners in diverse geographies.

A known limitation of this field study design for regulatory approval is the inherent difficulty in confirming individual enrolled dogs' exposure to infective mosquitoes. To support the assumption of exposure on a population level, the study was conducted across multiple heartworm-endemic and geographically varied sites, including sites in areas (LA, MS, NC, TN, TX) where resistant *D. immitis* isolates have been identified [38]. This site selection strategy was intended to increase the likelihood that the study population would be challenged and to enable evaluation of product performance in geographies that may harbor both susceptible and resistant heartworm populations. While these regions are considered endemic and represent field-based transmission scenarios, the authors note that county-level prevalence varied during the study period (Fig. 2) [37], suggesting that the intensity of the product challenge may have differed across certain locations. As is characteristic of this study framework, the focus remains on real-world efficacy rather than individual-level quantification of heartworm challenge. Within this context, the 100% efficacy achieved demonstrates that no failures occurred during the trial.

Macrocyclic lactones are the only drug class available and approved for prevention of heartworm disease [13]. Resistance to this class has been recognized in the USA [38–43]. As MLs are the only approved drug class for prevention of heartworm disease, the unique attributes of moxidectin's lipophilicity, high potency, and a long half-life may optimize efficacy against *D. immitis* and may contribute to overcoming the resistance mechanisms of *D. immitis* [13]*.* While the direct mechanism of action is unknown, the higher tissue distribution and longer half-life of moxidectin could allow for a greater effect on potential routes of action, including activity on excretory-secretory pores of the MF, hindering their ability to evade host defenses, as seen with *Brugia malayi* or through activity at GluCls binding sites [12].

Incorporating moxidectin into Credelio Quattro at a minimum dosage of 0.02 mg/kg provides effective heartworm prevention, as demonstrated by the two laboratory studies and clinical field study presented here. In the two laboratory studies, none of the 16 dogs administered IVP 30 days post-inoculation developed heartworm infections. In Study 2, dogs that were administered lotilaner alone developed heartworm infections comparable to those in the CP-treated group, demonstrating moxidectin is necessary in the combination tablet to prevent heartworm disease. The field study confirmed that Credelio Quattro provided heartworm protection under real-world conditions involving administration by pet owners.

The field study evaluated safety of > 1900 doses of Credelio Quattro, administered to 191 dogs. While reports of AEs are expected during a study of this duration, the most common AEs included transient gastrointestinal signs: diarrhea, with or without blood and vomiting, lethargy, anorexia, and dermatitis. None of the serious AEs reported in either treatment group were determined to be related to treatment. Credelio Quattro's safety profile and reported efficacy is consistent with the recommendations for monthly heartworm prevention from global veterinary organizations such as CAPC, the European Scientific Counsel Companion Animal Parasites (ESCCAP), and the Tropical Council for Companion Animal Parasites (TroCCAP). CAPC recommends year-round prevention for heartworm, intestinal parasites, and flea and tick control [16]. ESCCAP advises monthly treatment during the *D. immitis* vector season [44], while TroCCAP recommends year-round monthly prophylaxis for *D. immitis* [45]. Recommendations vary based on differences in climate and parasite distribution; therefore, veterinarians should educate pet owners on these recommendations and promote compliance with the appropriate strategic monitoring and control guidelines. Credelio Quattro can aid veterinarians, pet owners, and dogs by providing a single convenient monthly oral tablet designed to prevent heartworm disease caused by *D. immitis* and address the most common parasite risks.

## Conclusions

Credelio Quattro, administered monthly at the minimum dosages of 20 mg/kg lotilaner, 0.02 mg/kg moxidectin, 5.0 mg/kg praziquantel, and 5.0 mg/kg pyrantel, provided effective prevention of heartworm disease caused by *D. immitis* in dogs in laboratory studies. In a field study conducted in heartworm-endemic areas, no dog receiving Credelio Quattro tested positive for adult heartworm infection when dosed monthly for 11 consecutive doses. Credelio Quattro was well tolerated in all three studies.

## Data Availability

Data supporting the conclusions of this article are included within the article.

## Abbreviations

AE: Adverse event
AHS: American Heartworm Society
AM: Arithmetic mean
BW: Body weight
CAPC: Companion Animal Parasite Council
ESCCAP: European Scientific Counsel Companion Animal Parasites
L4: Fourth stage larvae
GM: Geometric mean
GA-3: Georgia III
GluCl: Glutamate-gate chloride ion channel
HW: Heartworm
LOE: Lack of effectiveness
ML: Macrocyclic lactone
MF: Microfilariae
CP: Negative control product
SP: Safety population
SAE: Serious adverse event
L3: Third-stage larvae
TroCCAP: Tropical Council for Companion Animal Parasites
USA: United States
